# Evolution of *Pfdhps* and *Pfdhfr* mutations before and after adopting seasonal malaria chemoprevention in Nanoro, Burkina Faso

**DOI:** 10.1038/s41598-024-75369-2

**Published:** 2024-10-16

**Authors:** Francis Emmanuel Towanou Bohissou, Paul Sondo, Juliana Inoue, Toussaint Rouamba, Berenger Kaboré, Guétawendé Job Wilfried Nassa, A. Elisée Sié Kambou, Tiampan Edwig Traoré, Victor Asua, Steffen Borrmann, Halidou Tinto, Jana Held

**Affiliations:** 1 Institute of Tropical Medicine, University Hospital Tübingen, Tübingen, Germany; 2 Institut de Recherche en Sciences de la Santé (IRSS)/Clinical Research Unit of Nanoro (CRUN), Nanoro, Burkina Faso; 3 Centre de Recherche Entomologique de Cotonou (CREC), Cotonou, Benin; 4 Infectious Diseases Research Collaboration, Kampala, Uganda; 5 German Center for Infection Research (DZIF), Partner Site Tübingen, Tübingen, Germany; 6 Centre de Recherches Médicales de Lambaréné, (CERMEL), Lambaréné, Gabon

**Keywords:** *Plasmodium falciparum*, *Pfdhfr*, *Pfdhps*, SMC, Burkina Faso, Genetic markers, Parasite genetics

## Abstract

Seasonal Malaria Chemoprevention consisting of monthly administration of amodiaquine/sulfadoxine-pyrimethamine to children aged 3–59 months during the transmission season could promote SP-resistance. Mutations in dihydrofolate reductase (*Pfdhfr*) and dihydropteroate synthase (*Pfdhps*) genes were assessed before and after SMC adoption in Burkina Faso. A total of 769 dried blood spots were selected from studies conducted in Nanoro, Burkina Faso, between 2010 and 2020. Of those, 299 were pre-SMC (2010–2012) and 470 were post-SMC-samples. *Pfdhps* and *Pfdhfr* genes were PCR-amplified and sequenced. A systematic review/meta-analysis of published studies conducted in Burkina Faso (2009–2023) was additionally performed. In Nanoro, the prevalence of *Pfdhfr* triple mutations (CIRNI) rose from 43.6% pre-SMC to 89.4% post-SMC (*p* < 0.0001). There was no mutation in *Pfdhfr* 164 and *Pfdhps* 540; *Pfdhps* A437**G** mutation increased from 63.9% (2010–2012) to 84.7% (2020) (*p* < 0.0001). The **VAG**K**GS** haplotype was 2.8% (2020). *Pfdhfr/Pfdhps* quintuple mutant **IRN**-436**A**437**G** rose from 18.6% (2010–2012) to 58.3% (2020) (*p* < 0.0001). Meta-analysis results from Burkina Faso showed an increase in mutations at *Pfdhfr* N51**I**, C59**R**, S108**N**, and *Pfdhps* A437**G** after SMC adoption. Post-SMC, the pyrimethamine-resistance marker prevalence increased, while the sulfadoxine-resistance marker prevalence remained stable. Detection of emerging *Pfdhps***VAG**K**GS** haplotypes in 2020 underscores the importance of continuous SP-resistance monitoring.

**Supplementary Information:**

The online version contains supplementary material available at 10.1038/s41598-024-75369-2.

## Introduction

Despite the implementation of various strategies to control malaria, it remains a significant health problem for endemic countries, particularly for children under five years and pregnant women. Malaria cases increased slightly from 244 million in 2021 to 249 million in 2022, resulting in 608,000 deaths^1^. Most of these cases and deaths occurred in countries within the World Health Organization (WHO) African Region, accounting for approximately 94% of global malaria cases and almost 96% of malaria-related deaths^1^. Importantly, 78.9% of the affected individuals were children under the age of five. Burkina Faso, a country where malaria transmission is seasonal, and where most cases are reported during the rainy season from June to November, contributed to 3.2% of global malaria cases^1^. In 2021, malaria accounted for 55.9% of hospitalizations and 15% of deaths nationwide, highlighting its substantial impact as the primary contributor to both hospital admissions and mortality rates in the country^2^.

In 2012, the WHO recommended the implementation of Seasonal Malaria Chemoprevention (SMC) in the Sahel sub-region of Africa, where malaria transmission occurs seasonally. SMC consists of monthly delivery of antimalarial drugs [amodiaquine/ sulfadoxine-pyrimethamine (SP)] to children aged 3–59 months during the 4-month of high malaria transmission season, annually, regardless of a child’s malaria status^3–5^. This intervention aims to clear malaria parasites and prevent new infections^3^. Several studies have shown that SMC can reduce malaria morbidity by 30–83%^6–9^. By 2021, 13 countries in the African Sahel region had implemented active SMC programs, treating approximately 45 million children per cycle^1^. This intervention has been implemented in Burkina Faso since 2014. By 2021, over 4 million children under the age of five have received four cycles of SMC treatment^2^. A study conducted by Kirakoya-Samadoulougou et al. demonstrated a 69% reduction of uncomplicated malaria incidence and 73% reduction of severe malaria incidence after two rounds of SMC conducted in 2015 and 2016 in the country^10^. Apart from SMC, the World Health Organization (WHO) recommends also intermittent preventive treatment with SP (IPTp-SP) to prevent malaria in pregnancy in areas of moderate-to-high transmission in sub-Saharan Africa. However, such intensive concomitant use of SP in the two strategies may spread resistance of *Plasmodium falciparum* malaria parasite to this drug, thereby reducing its efficacy.

Mutations of *Plasmodium falciparum* in target enzymes of SP, specifically dihydropteroate synthase (*Pfdhps*) and dihydrofolate reductase (*Pfdhfr*), are known to confer resistance to sulfadoxine and pyrimethamine, respectively. Five mutations in *Pfdhps* codons (S436**A/F**, A437**G**, K540**E**, A581**G**, and A613**S/T**) and five mutations in *Pfdhfr* codons (C50**R**, N51**I**, C59**R**, S108**N**, I164**L**) have been identified as associated with SP resistance^11–18^. The WHO recommends that countries withdraw IPTp-SP when the prevalence of mutations at *Pfdhps* K540**E** is > 95% and *Pfdhps* A581**G** is > 10% and withdraw intermittent preventive treatment in infancy (IPTi-SP) when the prevalence of *Pfdhps* K540**E** is > 50%^19–21^. In Africa, a variant known as **IRN-GE**, which includes mutations in both *Pfdhfr* (N51**I**, C59**R**, and S108**N** [**IRN**]) and *Pfdhps* (A437**G** and K540**E** [**GE**]), has been strongly linked to full SP resistance^22^, leading to clinical treatment failure^23–25^. Until recently, this **IRN-GE** mutant haplotype was relatively uncommon in West Africa compared to East and Southern Africa^26–30^.

A comparative analysis was conducted in seven West Sahel African countries (Burkina Faso, Chad, Guinea, Mali, Nigeria, Niger, and the Gambia) in 2016 and 2018 to assess the impact of scaling up SMC on SP resistance mutations. The study found a significant increase in the prevalence of the triple mutant *Pfdhfr ***IRN** two years after the implementation of SMC^31^. In contrast, the prevalence of *Pfdhps ***GE** remained low, affecting less than 1% of children under the age of five^31^. Similar trends were observed in other studies in Burkina Faso^30,32,33^. As the SMC program advances and the 2016 WHO guidelines recommend to increase the number of antenatal care (ANC) contacts to a minimum of eight with the aim to deliver a minimum of three or more doses of IPTp to women^34^, it becomes imperative to maintain a vigilant stance on its effectiveness by continually monitoring the development of *P. falciparum* resistance to SP. Examining the sustained impact of SMC on the emergence and progression of SP resistance is essential for ensuring the enduring effectiveness of this drug within the strategy. Understanding the evolution of these mutations following the adoption of SMC can provide valuable insights for shaping future malaria control policies and ensuring drug efficacy. Therefore, our study assessed the temporal trends of SP resistance markers from 2010 to 2020, both before and after the adoption of the SMC policy in Burkina Faso in 2014.

## Results

### Baseline characteristics of the study population

Seven hundred and sixty-nine (769) samples were randomly selected to analyse the *Pfdhfr* and *Pfdhps* mutations. Among them, 299 samples were collected between 2010 and 2012, and 240 and 230 were collected in 2018 and 2020, respectively. For 9/230 participants in 2020, the age was unknown and the samples were therefore excluded from the baseline description. The study population encompassed 74.6% (567/760) of children under five. Proportions of the latter derived from samples collected in 2010–2012, 2018, and 2020 were 73.6% (220/299), 56.3% (135/240), and 95.9% (212/221), respectively. Participants with symptomatic malaria represented 78.4% (596 out of 760) of the samples. The participant demographics, clinical status, and biological features are detailed in Table 1.

**Table 1:** Baseline characteristics of the study population from studies selected in 2010–2012 (before SMC), 2018 (4 years after SMC) and 2020 (6 years after SMC) in Nanoro, Burkina Faso.

	2010–2012 (*N* = 299)	2018 (*N* = 240)	2020 (*N* = 221)	Total (*N* = 760)
Age in years				
Mean	4.90 ± 6.26	6.09 ± 7.43	3.00 ± 1.04	4.72 ± 5.88
Median [Min, Max]	3.24 [0.241, 40.6]	4.19 [0.433, 60.1]	3.00 [0.600, 5.10]	3.31 [0.241, 60.1]
Sex				
Female	138 (46.2%)	131 (54.6%)	122 (55.2%)	391 (51.4%)
Male	161 (53.8%)	109 (45.4%)	99 (44.8%)	369 (48.6%)
Temperature				
Mean (SD)	38.5 (0.981)	38.2 (0.987)	36.8 (0.769)	37.9 (1.17)
Median [Min, Max]	38.5 [36.0, 41.0]	38.2 [36.0, 40.6]	36.7 [34.5, 38.9]	38.0 [34.5, 41.0]
Fever/History of fever				
No	0 (0%)	0 (0%)	164 (74.2%)	164 (21.6%)
Yes	299 (100%)	240 (100%)	57 (25.8%)	596 (78.4%)
Parasitemia				
Geometric mean	26,903.19	24,343.01	5166.74	15,725.38
Median [Min, Max]	31,400 [1880, 691,000]	42,500 [48.0, 564,000]	4860 [37.0, 390,000]	21,600 [37.0, 691,000]

### Temporal trends of *Pfdhfr*, *Pfdhps* and the combined *Pfdhfr/Pfdhps* genotypes

The sequencing success rate for the *Pfdhfr* gene was 96.9% (745/769), while that of the *Pfdhps* gene was 95.2% (732/769). The temporal trends of mutations at each codon and the associated haplotypes of *Pfdhfr* and *Pfdhps* genes were analysed by comparing the prevalence found in 2010–2012 (prior to the SMC adoption) with the prevalence found in 2018 (4 years after the SMC adoption) and 2020 (6 years after the SMC implementation).

**Fig. 1:**
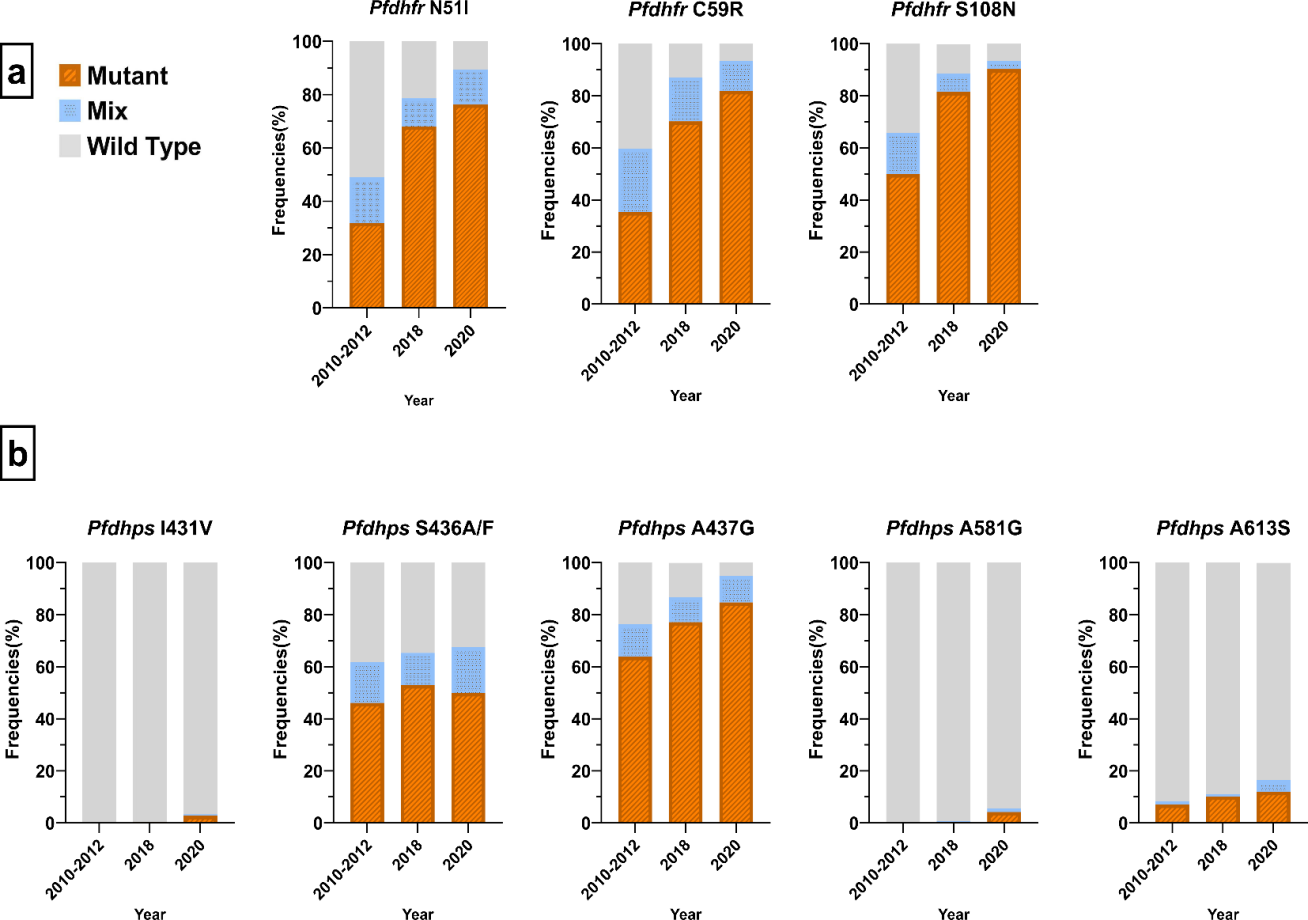
Temporal trends in prevalence of mutant, mixed and wild-type alleles of *Pfdhfr and Pfdhps* in 2010–2012, 2018 and 2020 in Nanoro, Burkina Faso. (**a**) *Pfdhfr* codons (2010–2012: *N* = 280; 2018: *N* = 238; 2020: *N* = 227); (**b**) *Pfdhps* codons (2010–2012: *N* = 280; 2018: *N* = 236; 2020: *N* = 216).

Except for the absence of mutations at *Pfdhfr* codons C50**R** and I164**L**, there was a significant increase (*p* < 0.0001 for each codon) of mutations observed at codons N51**I**, C59**R**, and S108**N** (Fig. 1 and Suppl. Table 1). The prevalence of the N51**I** mutation increased from 31.8% (89/280) before the SMC implementation (2010–2012) to 68.1% (162/238) in 2018 and climbed to 76.2% (173/227) in 2020. Similar trends were observed for codons C59**R** [35.4% (99/280) vs. 70.2% (167/238) vs. 81.9% (186/227)] and S108**N** [50.0% (140/280) vs. 81.5% (194/238) vs. 90.3% (205/227)]. The triple mutant *Pfdhfr* C**IRN**I at codons 51, 59, and 108 increased significantly from 43.6% in 2010–2012 (before the SMC implementation) to 77.3% in 2018 and 89.4% in 2020 (Table 2).

**Table 2:** Temporal trends in the prevalence of *Pfdhfr* and *Pfdhps* haplotypes in 2010–2012, 2018 and 2020 in Nanoro, Burkina Faso.

	Haplotypes	2010–2012 (before SMC) % (*n*/*N*)	2018 (4 years after SMC) % (*n*/*N*)	2020 (6 years after SMC) % (*n*/*N*)
*Pfdhfr*				
Wild type	C_50_N_51_C_59_S_108_I_164_	33.6 (94/280)	11.3 (27/238)	6.6 (15/227)
Single mutant	C**I**CSI	0.4 (1/280)	0 (0/238)	0 (0/227)
	CNC**N**I	2.5 (7/280)	0.4 (1/238)	0 (0/227)
Double mutant	C**I**C**N**I	3.9 (11/280)	1.3 (3/238)	0 (0/227)
	C**IR**SI	0.4 (1/280)	0 (0/238)	0 (0/227)
	CN**RN**I	15.7 (44/280)	9.7 (23/238)	4.0 (9/227)
Triple mutant	C**IRN**I	43.6 (122/280)	77.3 (184/238)	89.4 (203/227)
*Pfdhps*				
Wild type	I_431_S_436_A_437_K_540_A_581_A_613_	5.0 (14/280)	1.7 (4/236)	0 (0/216)
Single mutant	I**A**AKAA	15.7 (44/280)	8.5 (20/236)	2.3 (5/216)
	IS**G**KAA	30.4 (85/280)	30.5 (72/236)	30.6 (66/216)
	ISAKA**S**	1.4 (4/280)	1.3 (3/236)	0.9 (2/216)
Double mutant	I**A**AKA**S**	1.1 (3/280)	0.8 (2/236)	0 (0/216)
	I**FG**KAA	0.4 (1/280)	0.4 (1/236)	0 (0/216)
	I**F**AKA**S**	0.4 (1/280)	0.4 (1/236)	0.9 (2/216)
	I**AG**KAA	40.4 (113/280)	47.0 (111/236)	49.5 (107/216)
	IS**G**KA**S**	1.1 (3/280)	1.3 (3/236)	0 (0/216)
	ISAK**GS**	0 (0/280)	0 (0/236)	0.9 (2/216)
Triple mutant	I**A**AK**GS**	0 (0/280)	0.4 (1/236)	0 (0/216)
	I**AG**KA**S**	4.3 (12/280)	7.2 (17/236)	9.3 (20/216)
	I**AG**K**G**A	0 (0/280)	0 (0/236)	0.9 (2/216)
	I**FG**KA**S**	0 (0/280)	0 (0/236)	0.5 (1/216)
Quadruple mutant	I**AG**K**GS**	0 (0/280)	0 (0/236)	0.9 (2/216)
	**VAG**KA**S**	0 (0/280)	0 (0/236)	0.5 (1/216)
Quintuple mutant	**VAG**K**GS**	0 (0/280)	0.4 (1/236)	2.8 (6/216)
*Pfdhfr/Pfdhps*				
Quadruple mutant	**IRN-436 A**	26.8 (72/269)	50.6 (119/235)	60.2 (130/216)
	**IRN-437G**	32.0 (86/269)	68.9 (162/235)	85.2 (184/216)
	**IRN-581G**	0 (0/269)	0 (0/235)	5.6 (12/216)
Quintuple mutant	**IRN-436A437G**	18.6 (50/269)	43.8 (103/235)	58.3 (126/216)
	**IRN-436A581G**	0 (0/269)	0 (0/235)	4.6 (10/216)
	**IRN-437G581G**	0 (0/269)	0 (0/235)	4.6 (10/216)
Octuple mutant	C**IRN**I**-VAG**K**GS**	0 (0/269)	0 (0/235)	2.8 (6/216)
Wild type	**Wild Type**	0.4 (1/269)	0.4 (1/235)	0 (0/216)

The mutation patterns within the *Pfdhps* gene exhibited varying trends across specific codons (Fig. 1 and Suppl. Table 2). No mutations were detected at *Pfdhps* K540**E** as all codons were wild type at this position. However, there was a significant increase (*p* < 0.0001) in the occurrence of mutations at codon A437**G**, escalating from 63.9% (179/280) in 2010–2012 to 77.1% (182/236) in 2018 and further climbing to 84.7% (183/216) in 2020. A similar upward trend was observed at codon *Pfdhps* A613**S**, with frequencies of 7.1% (20/280) in 2010–2012, 10.2% (24/236) in 2018, and 12.0% (26/216) in 2020. In contrast, mutations reported at codon S436**A** maintained a consistent presence over time: 46.1% (129/280) in 2010–2012, 53.0% (125/236) in 2018, and 49.5% (107/216) in 2020. Mutations at codons I431**V** and A581**G** emerged exclusively after the SMC implementation, reaching 2.8% (6/216) and 4.2% (9/216) in 2018 and 2020, respectively.

Analysis of the combination at codons 431, 436, 437, 540, 581, and 613 within *Pfdhps* revealed the presence of 17 distinct haplotypes. The prevalence of the single mutant *Pfdhps* haplotype, IS**G**KAA, remained steady at approximately 30% before and after SMC implementation. Conversely, double mutant haplotypes associated with mutations at codons 436 and 437, specifically I**AG**KAA, increased from 40.4% in 2010–2012 to 47.0% in 2018, further to 49.5% in 2020 (*p* = 0.0374). The parasites harbouring the *Pfdhps* haplotype with at least the triple mutation at codons 436, 437 and 581 were predominantly observed in 2020 and accounted for 4.6% (10/216). Similarly, quintuple mutant **VAG**K**GS** was only detected in the post-SMC period at 2.8% (6/216) in 2020 (Table 2).

For the combined *Pfdhfr/Pfdhps* genotypes, from the 41 haplotypes identified, the prevalence of parasites with at least the quadruple mutant **IRN**-437**G** notably surged following SMC implementation, increasing from 27.1% in 2010–2012 to 51.1% in 2018 and stabilizing at 58.3% in 2020. The occurrence of the quintuple mutations **IRN**-436**A**581**G** and IRN-437**G**581**G** was exclusively documented in 2020, accounting for 10.6% (23/216) and 0.9% (2/216), respectively. Furthermore, in 2020, the octuple mutant C**IRN**I/**VAG**K**GS** was present at 2.8% (6/216), (Table 2 and Suppl. Table 3).

### Trends of SP resistance in Burkina Faso based on the data from previous studies conducted between 2009 and 2023

**Fig. 2:**
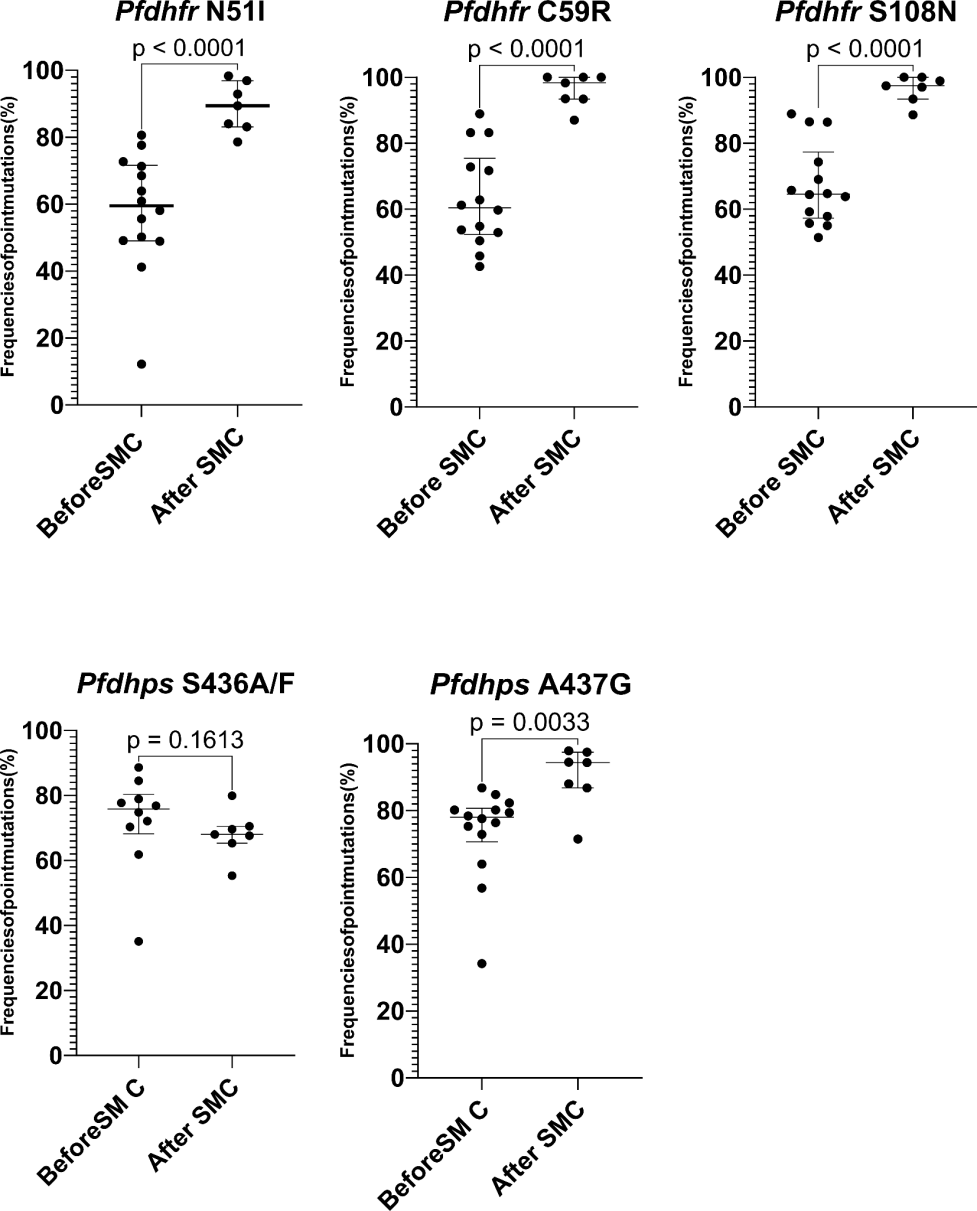
Prevalence of mutations at codons *Pfdhfr* and *Pfdhps* from previous studies conducted between 2009 and 2023 in Burkina before and after implementing SMC. The prevalence rates of various mutations documented in each study were graphed and illustrated by individual data points. Two distinct groups were delineated: one comprising studies conducted before the implementation of SMC (before 2014) and the other encompassing studies conducted after the adoption of SMC (after 2014). Each group’s median (with the interquartile) was compared using a non-parametric test (Wilcoxon Mann–Whitney test).

To get a broader picture of the trends of SP resistance mutations before and after SMC implementation in Burkina Faso, we additionally looked at previously published data and included them in our analysis. Figure 2 illustrates a compilation of published data from studies conducted from 2009 to 2023 in Burkina Faso, showcasing the median and interquartile ranges of the prevalences of selected mutations before and after the SMC implementation. In total, eleven (11) studies (10 from previous studies and one from our current analysis conducted in Nanoro) were included (Suppl. Table 4). Heterogeneity between studies was assessed using Cochran’s Q Test and the I² Statistic within the random-effects model. No significant heterogeneity was observed that could impact the meta-analysis (Suppl. Table 5). These studies reported an increase in the prevalence of mutations at *Pfdhfr* codons 51, 59, and 108 following the implementation of SMC. The prevalence ranged from 12.2 to 86.4% before SMC, escalating to 78.6–100% after its implementation. Similarly, the triple mutant haplotype *Pfdhfr***IRN** increased from 11.4 to 73.9% before SMC to 73.9–98.1% after SMC.

Regarding *Pfdhps* A437**G** mutations, before the introduction of SMC, its prevalence spanned from 34.2 to 86.8%. Following the implementation of SMC, a significant increase was observed, with prevalence ranging from 86.8 to 94.5%. Conversely, the prevalence of mutations at *Pfdhps* codons S436**A** remained consistent irrespective of SMC implementation, typically fluctuating between 50% and 80%. Of note, our study is the first to report mutations at *Pfdhps* codons I431**V** in Burkina Faso (1 case in 2018 and 7 cases in 2020). However, the quintuple mutant **IRN-GE** remained relatively rare at around 1%.

### Linkage disequilibrium (LD) analysis

**Fig. 3:**
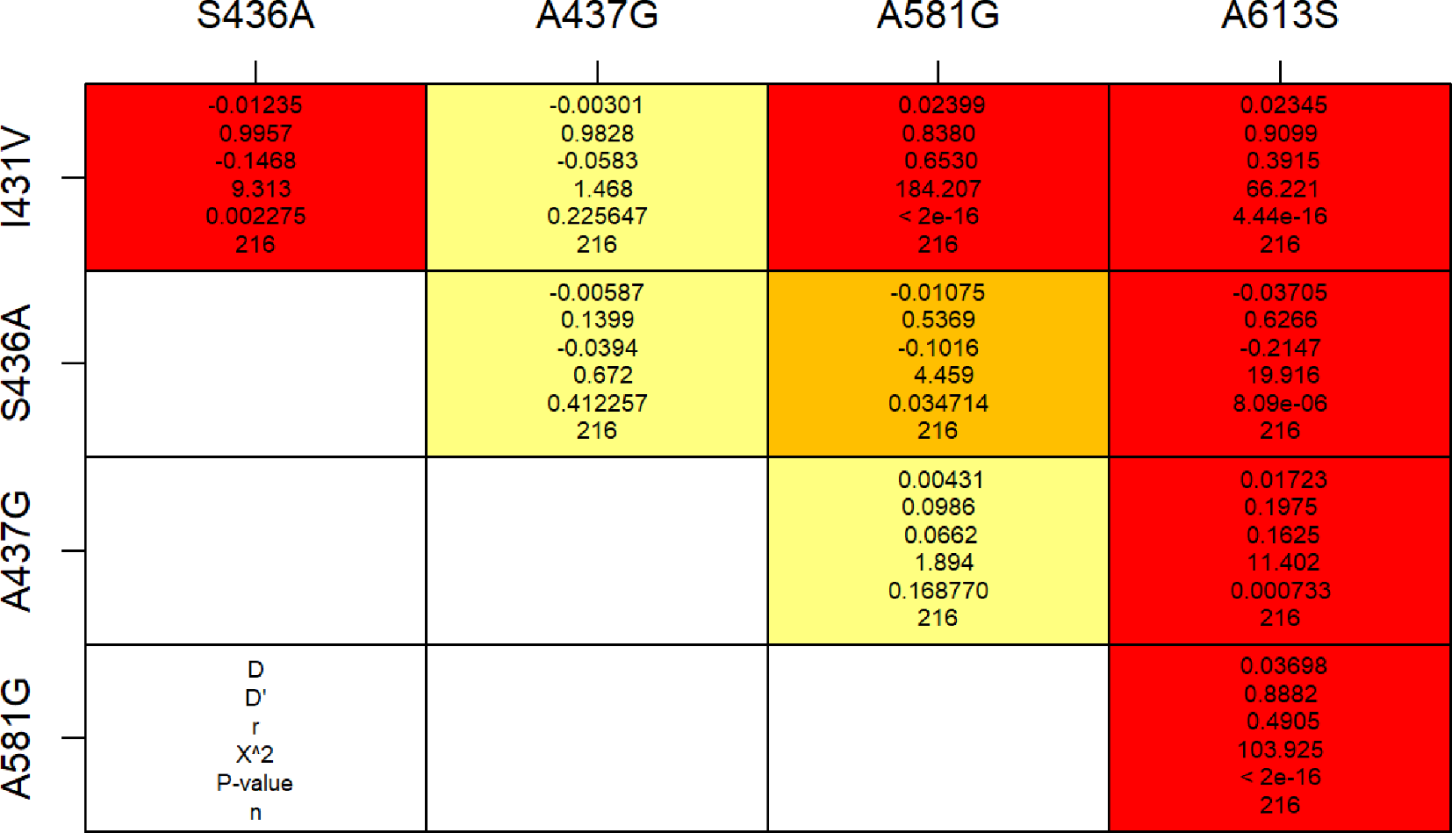
Linkage disequilibrium (LD) analysis of *Pfdhps* SNPs related to the mutations I431**V**, S436**A**, A437**G**, A581**G** and A613**S**. The dark red indicates a strong statistically significant (*p* < 0.001) linkage. Yellow indicates that no linkage is present. Orange indicates the presence of the linkage with a statistically significant (*p* < 0.05) linkage.

Figure 3 presents the pairwise linkage disequilibrium of alleles at codon I431**V** with others in *Pfdhps* from the samples selected in 2020. Significant statistical associations were observed between the alleles at codon I431**V** and alleles at codons S436**A** (D’=0.996, *p* = 0.0022), A581**G** (D’=0.838, *p* < 0.0001), and A613**S** (D’=0.909, *p* < 0.0001).

## Discussion

Our study revealed an uptick in mutations in the *Pfdhfr* gene, linked explicitly to pyrimethamine resistance following the SMC policy implementation in Burkina Faso. Conversely, the prevalence of mutations associated with sulfadoxine resistance within the *Pfdhps* gene remained relatively constant. These results align with similar conclusions drawn from a study conducted in Burkina Faso and six other sub-Saharan African countries (The Gambia, Guinea, Mali, Niger, Nigeria, and Chad) by Beshir et al., in 2018, two years after the expansion of the SMC in the Sahel region^31^. The latter focused on two age groups—children under five years of age and individuals aged 10–30 years — highlighting the widespread presence of the *Pfdhfr ***IRN** haplotype at codons 51, 59, and 108 across all study sites. Like our findings, the allele associated with high-level resistance due to an additional mutation at codon 164 was not reported. Beshir et al., also showed significant variability in the *Pfdhps* gene, displaying the intricate patterns of amino acid substitution with a description of novel mutations^31^. Despite the substantial diversity observed within *Pfdhps* haplotypes, no new mutations were observed in our study. The difference observed between the increase in mutations in the *Pfdhfr* gene, and the general stability in the *Pfdhps* genes could be explained by several factors. Parasite drug resistance is affected by the parasite’s mutation rate, the fitness costs linked to the mutation, the overall parasite burden, and treatment adherence^35^. The differential selection pressures exerted by sulfadoxine and pyrimethamine could result in varying rates of mutations in the genes *Pfdhps* and *Pfdhfr*, respectively. However, the fitness of SP-resistant parasites in *Plasmodium falciparum* remains inconclusive. This is due to the resistance being driven by a complex combination of mutations in *Pfdhps* and *Pfdhfr*, making it challenging to independently evaluate the extent and impact of the fitness costs associated with mutations in *Pfdhps* and *Pfdhfr*^36–38^.

Our study reported a notable increase in the prevalence of the *Pfdhps* A437**G** mutation either individually or in combination with S436**A**, leading to the formation of I**AG**KAA alleles after the SMC implementation. Moreover, there was an increase in *Pfdhfr/Pfdhps* haplotypes, particularly **IRN**-437**G** or **IRN**-436**A**437**G**, which is described as conferring the first-level resistance to SP^19^. However, this does not affect the protective effect of SMC and intermittent preventive treatment during pregnancy (IPTp) concerning low birth weight^39^.

In contrast, the supplementary *Pfdhps* K540**E** mutation was reported to decrease the efficacy of both IPTp and SMC^40,41^, and the additional *Pfdhps* A581**G** mutation further exacerbates this resistance^5,39^. A loss of IPTp-SP effectiveness was reported when K540**E** prevalence surpassed 90%, and A581**G** exceeded 10%^39^. Consequently, WHO does not recommend the implemention of SMC in regions where the prevalence of the *Pfdhps* K540**E** mutation exceeds 50%^3^. Although the prevalence of these mutations has remained relatively low in our study and has never concurrently occurred with A581**G**, the latter has notably increased since the implementation of SMC but remained below 10%. Therefore, there is currently no immediate threat to the effectiveness of SP in Burkina Faso. However, to be prepared, and given the observed trends in SP resistance, alternative strategies should be explored. This could include community-based intervention for malaria prevention as potential approach^42^. Additionally, evaluating new antimalarial drugs like dihydroartemisinin-piperaquine, previously tested in many studies for preventing malaria in pregnant women^43–45^, may offer another option. It is also imperative that the National Malaria Control Program supports systematic surveillance of SP resistance mutations and rigorously monitors the efficacy of SP in the context of both SMC and prevention in pregnant women.

A matter of significant concern was the emergence of *Pfdhps* haplotypes characterised by various combinations of mutations involving I431**V**, S436**A**, and A581**G**, forming patterns such as **VAG**K**GS** or **VAG**KA**S**. The latter was first documented previously in 2007 in Nigeria^28,46^. The identification of the new mutation (I431**V**) has already been reported in neighbouring countries of Burkina Faso in West Africa, including Benin, Niger, Mali, Ghana, and Côte d’Ivoire^47^. Furthermore, this mutation has been identified in several other Central African countries, such as Cameroon, Chad, the Republic of Congo, Gabon, the Central African Republic, and the Democratic Republic of Congo^47^. Thus, this discovery emphasises the critical necessity for a continuous surveillance within specific regions and across borders to monitor and understand the spread of drug-resistant malaria strains.

Although the exact impact of these newly identified mutations remains unknown, the presence of the C**IRN**I/**VAG**K**GS** haplotypes, notably observed in a study among IPTp-SP pregnant women in Cameroon compared to non-SP recipients, suggests a possible association of this mutation with resistance to SP^48^. Moreover, despite the absence of clear block transmission evidence, the linkage disequilibrium analysis showed a robust association between this mutation and others at the *Pfdhps* gene, indicating either close physical proximity or a functional relationship. Guémas et *al*., upon investigating the extended haplotype homozygosity around the *Pfdhps* gene, focusing on the I431**V** mutation, reported that parasites carrying this mutation evolve under positive selection^47^. Consequently, parasites with the I431**V** mutation in the *Pfdhps* gene seem to gain a selective advantage, hinting that this mutation provides some benefit or advantage to these parasites in their environment. Overall, the parasites carrying this mutation evolve due to positive selection, potentially implying an advantageous trait linked to this genetic change for their survival or reproductive success.

The absence of this mutation in East Africa, where there is a high prevalence of mutations at K540**E**, leads to the speculation that I431**V** may hold a similar significance in West and Central Africa as K540**E** does in East Africa—an indicator of high resistance to SP^47^. Further research should focus on the impact of this mutation on SP efficacy for both IPTp and SMC. Additionally, a long-term follow-up could be a valuable approach to further characterize the mutations associated with SP resistance following SMC implementation and assessing their impact on the drug’s efficacy.

In this study, not all the samples from the included studies were genotyped; they were only randomly selected samples, increasing the risk of missing rare SNPs and haplotypes, and this is one limitation. In addition, the last samples were collected in December 2020; continuous sampling and analysis should be implemented to know the current situation.Expanding the number of time points and including multiple study sites across Burkina Faso would have better captured the SP resistance trends. Furthermore, considering the utilisation of SP in combination with amodiaquine for SMC, additionally analyzing mutations at *Pfcrt* would provide valuable insights.

In conclusion, this study offers a significant understanding of the dynamics of SP resistance in Burkina Faso across a decade. It is (at our knowledge) the first study in the study area to investigate the link between SMC and SP resistance across three different time points. Following the implementation of SMC, there was a noteworthy increase in the prevalence of pyrimethamine resistance markers, while no discernible differences were observed in sulfadoxine resistance markers. Nonetheless, SP remains a viable option for malaria prophylaxis in pregnant women and children. Identifying the emerging *Pfdhps mutant* haplotypes **VAG**K**GS** in 2020 underscores the need to monitor SP resistance continuously. Despite WHO recommendations, the absence of systematic monitoring in the country underscores the need to establish a resistance surveillance system at designated sentinel sites. Furthermore, policymakers should promote research initiatives that foster creativity and innovation to develop novel strategies to combat resistance. Therefore additional research is necessary to understand how mutations could affect the effectiveness of sulfadoxine-pyrimethamine in preventing malaria in children and during pregnancy. Eliminating malaria remains a significant challenge, particularly given the growing resistance to drugs like SP. Achieving this goal requires a comprehensive approach that includes monitoring and anticipating antimalarial drug resistance.

## Methods

### Study site

Samples analysed in this study were collected from individuals living in Nanoro Health District (NHD) in Burkina Faso, West Africa. NHD is located in a rural setting in the country’s central-western part, at approximately 85 km from Ouagadougou, the capital city^49^. Malaria transmission is endemic in the region and follows a distinct seasonal pattern. The year-round transmission persists, peaking significantly from July to December, aligning with the rainy season. *P. falciparum* is the most prevalent species associated with malaria infections. SMC was adopted in Burkina Faso in 2014 and has been implemented in this area since 2016 in addition to IPTp-SP^49,50^.

### Selected studies and samples

A total of 769 samples tested positive for *Plasmodium falciparum* by microscopy were randomly chosen from previous studies conducted in the NHD between 2010 and 2020, pre- and post-SMC adoption (2014). All samples analysed were previously collected on dried blood spots and stored in a temperature-controlled environment under consistent conditions. Before SMC implementation in 2014, we selected 299 samples (from 680 collected) from 2010 to 2012. After the introduction of SMC, samples from two additional periods were included: 240 samples (from a total of 549) from 2018 and 230 samples (from a total of 298) from 2020. These samples encompass three separate studies: one was conducted before the adoption of SMC, while the other two were carried out four and six years after its implementation.

The first study was a pharmacovigilance project conducted from 2010 to 2012 (before the SMC implementation). It assessed the safety and effectiveness of artesunate-amodiaquine (ASAQ) versus artemether-lumefantrine (AL) for treating uncomplicated malaria. The trial protocol was registered on ClinicalTrials.gov NCT01232530, and the results have been published elsewhere^51^. Briefly, all patients over six (6) months presenting uncomplicated falciparum malaria were enrolled and randomly assigned to receive either AL or ASAQ. They were followed up for 28 days. For our analysis, we selected dried blood spots (DBS) collected at Day 0 (before treatment) and positive for *Plasmodium* infection by microscopy. A total of 299 dried blood spots were selected from the participants enrolled from 2010 to 2012, representing the set of samples collected before the implementation of SMC.

The second study was a phase IV, observational, non-comparative study that assessed the clinical safety of Pyramax^®^ (Pyronaridine-Artesunate) administered to patients under real life conditions of antimalarial use. The study included all participants (all ages with weight ≥ 5 kg) attending the health centre with suspicion of uncomplicated malaria. The study was conducted in 2018 (four years after SMC implementation), and the methodology was described elsewhere^52^. From this study, 240 DBS were randomly selected from microscopically *P. falciparum* positive patients at Day 0 (before treatment).

The third study was a randomised open-label trial conducted in 2020 (six years after the SMC implementation). Children (6–59 months) under SMC coverage receiving vitamin A supplementation were randomly assigned to one of the following three study arms: (a) SMC + vitamin A alone, (b) SMC + vitamin A + zinc, or (c) SMC + vitamin A + Plumpy’Doz™. The trial was registered under ClinicalTrials.gov (NCT04238845) and published elsewhere^53^. We have randomly selected 230 DBS from microscopically *P. falciparum* positive patients from December 2020.

### Ethics statement

This study was approved by the ethical commission of the Faculty of Medicine of the University of Tübingen, Germany (Project 704/2022BO2). Ethical approval was provided for all previous studies, and written informed consent was obtained from each participant for further investigations on the parasite.

All methods were performed in accordance with the relevant guidelines and regulations.

### Sample processing and genotyping of *Pfdhfr* and *Pfdhps* genes

DNA was extracted from DBS using the QIAamp^®^ Blood mini kit (Qiagen GmbH., Hilden, GERMANY) per the manufacturer’s recommendations. Extracted DNA was stored at − 20 °C until further use.

The *Pfdhfr* and *Pfdhps* gene fragments covering the regions of interest were amplified by nested polymerase chain reaction (PCR). Outer (nest 1) and inner (nest 2) primers of *Pfdhps* used were those described in Duraisingh et al.^54^ and Kanai et al.^55^, respectively. Outer *Pfdhfr* primer was reported by Crameri et al.^56^. Considering the potential DNA degradation in samples from 2010 to 2012, tailored outer and inner primers were synthesized to streamline their analytical processing by partitioning the *Pfdhps* gene sequences of interest into two distinct fragments (205 bp and 540 bp). The first fragment encompassed codons 431, 436, and 437, while the second included codons 540, 581, and 613. The semi-nested PCR was performed using the same PCR reaction and cycling conditions. All designed primers are presented in Table 3. The same primers were employed for sequencing.

**Table 3:** Primers used for this study.

Primers	Sequences	Amplicon sizes (bp)
Nest 1 forward *dhfr*	5′-ACAAGTCTGCGACGTTTTCGATATTTATG-3′	646
Nest 1 reverse *dhfr*	5′-AGTATATACATCGCTAACAGA-3′	
Nest 2 forward *dhfr*	5′-GTGCATGTTGTAAGGTTGAAAGC-3′	542
Nest 2 reverse *dhfr*	5′-TACATCACATTCATATGTACTAT-3′	
Nest 1 forward *dhps*	5′-ATGCTTAAATGATATGATACCCG-3′	970
Nest 1 reverse *dhps*	5′-CATGTAATTCATCTGAAACATCC-3′	
Nest 2 forward *dhps*	5′-AACCTAAACGTGCTGTTCAA-3′	708
Nest 2 reverse *dhps*	5′-AATTGTGTGATTTGTCCACAA-3′	
Nest 1 forward *dhps fragment 1*	5′- TGTTGAACCTAAACGTGCTGT-3′	634
Nest 1 reverse *dhps fragment 1*	5′- TTGATCATTCATGCAATGGGC-3′	
Nest 2 reverse *dhps fragment 1*	5′- TGGTTTCGCATCACATTTAACA-3′	208
Nest 1 forward *dhps fragment 2*	5′- GAGAATCCTCTGGTCCTTTTGT-3′	645
Nest 1 reverse *dhps fragment 2*	5′- TCCAATTGTGTGATTTGTCCACA-3′	
Nest 2 forward *dhps fragment 2*	5′- TGTTAAATGTGATGCGAAACCA-3′	533

The nest 2 *Pfdhfr* PCR reaction was composed of 0.5 µl of nest 1 product, 1× PCR Buffer, 0.1 µl Taq DNA Polymerase, 0.25 mM of dNTP and 0.3 µM of each primer. All PCR ingredients were from Qiagen (Hilden, Germany). For nest 2, the PCR cycling conditions were 95 °C for 3 min, initial denaturation succeeded by 30 cycles of 95 °C for 30 s, 56 °C for 30 s, and 72 °C for 1 min. Positive (*P. falciparum* 3D7 and Dd2) and negative controls (uninfected erythrocytes and water) were included in each PCR.

The PCR products were revealed by using QIAxcel^R^ advanced (QIAGEN). Clear bands of the correct size were purified with ExoSAP-IT™ Express PCR Product Cleanup Reagent (Thermo Fisher Scientific Inc, USA) before being sent to Eurofins Genomics (Germany GmbH, Ebersberg Germany) for Sanger sequencing with the corresponding nest 2 primers.

### Data analysis

Sequencing results were analysed with Geneious Prime^®^ 2023.0.4 (Biomatters, San Francisco, CA, USA) using *P. falciparum* 3D7 reference genes, particularly PF3D7_0417200 and PF3D7_0810800 for *Pfdhfr* and *Pfdhps* genes, respectively. Sequences of poor quality (scores < 10%) were discarded, and samples were resent for sequencing. After cleaning the raw data, only the high-quality sequences (scores superior to 50%) were kept for analysis. For each sample, sequences were compared to reference sequence genome 3D7. Mutations were detected by visualising the mismatched alignment between nucleotides at different codons of interest on the gene. The presence of mixed infection was deduced for a sample if the sequencing chromatogram exhibited evenly sized, dual peaks across the examined locus.

The mutations investigated in *Pfdhfr* were C50**R**, N51**I**, C59**R**, S108**N**, and I164**L** (amino acid substitutions in boldface). For *Pfdhps*, the mutations I431**V**, S436**A/F**, A437**G**, K540**E**, A581**G**, and A613**S/T** were analysed. The prevalence of different alleles and haplotypes in the *Pfdhfr* and *Pfdhps* genes was reported, especially the allele as wild type (having only the wild type allele), mutant (having only the mutant allele), or mixed infection (having both wild type and mutant alleles). In the final analysis for describing haplotypes, mixed infections were considered mutants.

The software R (Rstudio version 4.2.2; R Core Team (2023) from R Foundation for Statistical Computing, Vienna, Austria < https://www.R-project.org/>) was used for statistical analysis. The proportions were compared using the appropriate χ2 (or Cochran Armitage test) for analysing the temporal trends in the prevalence of mutations. Bonferroni correction was applied to correct for multiple comparisons. Moreover, the linkage disequilibrium between the mutation I431**V** and other mutations of the *Pfdhps* was assessed using the package “genetics”. All statistical analyses were performed at the 5% significance level. Prism 10.0 software (GraphPad Software, San Diego, California, USA) was used for complementary analysis of the meta-analysis and graphs.

### Collection of historical data

To support the findings of our study, a systematic review and meta-analysis were performed on genotypic data of *P. falciparum* from studies conducted throughout Burkina Faso. Specifically, scientific articles focusing on the prevalence of SP molecular markers of resistance in Burkina Faso between 2009 and 2023 were sought from databases including Pubmed and Scopus. Eligible studies encompassed analyses of the genes *Pfdhfr* and *Pfdhps*, with sample collection from 2009 to 2023 based on the following quality assessment criteria: samples were collected before any treatment, the methods used to identify mutations were clearly described, and the prevalence of mutations at each codon of interest was reported. The prevalence of mutations at individual codons of interest within *Pfdhps* (S436**A/F**, A437**G**, K540**E**, A581**G**, and A613**S/T)** and *Pfdhfr (*C50**R**, N51**I**, C59**R**, S108**N**, and I164**L**) was extracted and subjected to analysis. A comprehensive comparative analysis was subsequently conducted to discern shifts in mutation prevalence at specific codons and haplotypes within the *Pfdhfr* and *Pfdhps* genes before and after the implementation of SMC. Mutation prevalence data from each study were stratified into two distinct temporal cohorts delineated by the period preceding and succeeding the implementation of SMC in 2014. The median prevalence of mutations at each codon within each temporal cohort was then carefully compared via a non-parametric test (Wilcoxon Mann-Whitney test) for statistical analysis.

## Electronic supplementary material

Below is the link to the electronic supplementary material.

Supplementary Material 1

## Data Availability

The datasets generated during and/or analyses during the current study are available from the corresponding author upon request.
