# Supplementary material for: Evolution of Pfdhps and Pfdhfr mutations before and after adopting seasonal malaria chemoprevention in Nanoro, Burkina Faso
Source: Sci Rep. 2024 Oct 16;14:24224. doi: 10.1038/s41598-024-75369-2 (PMC11484836; doi:10.1038/s41598-024-75369-2)
Supplement: Supplementary file 1 — Supplementary Material 1 [file 41598_2024_75369_MOESM1_ESM.pdf]

## SUPPLEMENTARY INFORMATION

# Evolution of *Pfdhps* and *Pfdhfr* mutations before and after adopting seasonal malaria chemoprevention in Nanoro, Burkina Faso


### Authors

Francis Emmanuel Towanou Bohissou[1,2,3], Paul Sondo[2], Juliana Inoue[1], Toussaint Rouamba[2], Berenger Kaboré[2], Guétawendé Job Wilfried Nassa[2], Sié A. Elisée Kambou[2], Tiampan Edwig Traoré[2], Victor Asua[1,4] , Steffen Borrmann[1,6], Halidou Tinto[2*], Jana Held[1,5,6*]

### Affiliations

1. Institute of Tropical Medicine, University Hospital Tübingen, Germany.
2. Institut de Recherche en Sciences de la Santé (IRSS)/Clinical Research Unit of Nanoro (CRUN), Nanoro, Burkina Faso.
3. Centre de Recherche Entomologique de Cotonou (CREC), Cotonou, Benin.
4. Infectious Diseases Research Collaboration, Kampala, Uganda.
5. German Center for Infection Research (DZIF), Partner Site Tübingen, Tübingen, Germany
6. Centre de Recherches Médicales de Lambaréné, (CERMEL), Lambaréné, Gabon

Corresponding authors:

*Jana Held : jana.held@uni-tuebingen.de

*Halidou Tinto : halidoutinto@gmail.com

**Supplementary Table 1:** Trends of *Pfdhfr* codons from 2010 to 2020 in Nanoro, Burkina Faso. The number of wild-type, mixed infection, and mutant alleles and their respective percentages are presented for each time point.

| | 2010-2012 (N=280) | 2018 (N=238) | 2020 (N=227) | Total (N=745) |
|---|---|---|---|---|
| **C50R** | | | | |
| Wild Type | 280 (100%) | 238 (100%) | 227 (100%) | 745 (100%) |
| **N51I** | | | | |
| Mix | 48 (17.1%) | 25 (10.5%) | 30 (13.2%) | 103 (13.8%) |
| Mutant | 89 (31.8%) | 162 (68.1%) | 173 (76.2%) | 424 (56.9%) |
| Wild Type | 143 (51.1%) | 51 (21.4%) | 24 (10.6%) | 218 (29.3%) |
| **C59R** | | | | |
| Mix | 68 (24.3%) | 40 (16.8%) | 26 (11.5%) | 134 (18.0%) |
| Mutant | 99 (35.4%) | 167 (70.2%) | 186 (81.9%) | 452 (60.7%) |
| Wild Type | 113 (40.4%) | 31 (13.0%) | 15 (6.6%) | 159 (21.3%) |
| **S108N** | | | | |
| Mix | 44 (15.7%) | 17 (7.1%) | 7 (3.1%) | 68 (9.1%) |
| Mutant | 140 (50.0%) | 194 (81.5%) | 205 (90.3%) | 539 (72.3%) |
| Wild Type | 96 (34.3%) | 27 (11.3%) | 15 (6.6%) | 138 (18.5%) |
| **I164L** | | | | |
| Wild Type | 280 (100%) | 238 (100%) | 227 (100%) | 745 (100%) |

**Supplementary Table 2:** Trends of *Pfdhps* codons from 2010 to 2020 in Nanoro, Burkina Faso. The number of wild-type, mixed infection and mutant alleles and their respective percentages are presented for each time point.

| | 2010-2012 (N=280) | 2018 (N=236) | 2020 (N=216) | Total (N=732) |
|---|---|---|---|---|
| **I431V** | | | | |
| Mix | 0 (0%) | 0 (0%) | 1 (0.5%) | 1 (0.1%) |

| | 2010-2012 (N=280) | 2018 (N=236) | 2020 (N=216) | Total (N=732) |
|---|---|---|---|---|
| Mutant | 0 (0%) | 1 (0.4%) | 6 (2.8%) | 7 (1.0%) |
| Wild Type | 280 (100%) | 235 (99.6%) | 209 (96.8%) | 724 (98.9%) |
| **S436A/F** | | | | |
| Mix | 44 (15.7%) | 29 (12.3%) | 39 (18.1%) | 112 (15.3%) |
| Mutant | 129 (46.1%) | 125 (53.0%) | 107 (49.5%) | 361 (49.3%) |
| Wild Type | 107 (38.2%) | 82 (34.7%) | 70 (32.4%) | 259 (35.4%) |
| **A437G** | | | | |
| Mix | 35 (12.5%) | 23 (9.7%) | 22 (10.2%) | 80 (10.9%) |
| Mutant | 179 (63.9%) | 182 (77.1%) | 183 (84.7%) | 544 (74.3%) |
| Wild Type | 66 (23.6%) | 31 (13.1%) | 11 (5.1%) | 108 (14.8%) |
| **K540E** | | | | |
| Wild Type | 280 (100%) | 236 (100%) | 216 (100%) | 732 (100%) |
| **A581G** | | | | |
| Mix | 0 (0%) | 1 (0.4%) | 3 (1.4%) | 4 (0.5%) |
| Mutant | 0 (0%) | 1 (0.4%) | 9 (4.2%) | 10 (1.4%) |
| Wild Type | 280 (100%) | 234 (99.2%) | 204 (94.4%) | 718 (98.1%) |
| **A613S** | | | | |
| Mix | 3 (1.1%) | 2 (0.8%) | 10 (4.6%) | 15 (2.0%) |
| Mutant | 20 (7.1%) | 24 (10.2%) | 26 (12.0%) | 70 (9.6%) |
| Wild Type | 257 (91.8%) | 210 (89.0%) | 180 (83.3%) | 647 (88.4%) |

**Supplementary Table 3:** Trends of combined haplotypes *Pfdhfr/Pfdhps* from 2010 to 2020 in Nanoro, Burkina Faso. The number of the combined haplotypes and their respective percentages are presented for each time point.

| *Pfdhfr/Pfdhps* | 2010-2012 (N=269) | 2018 (N=235) | 2020 (N=216) | Total (N=720) |
|---|---|---|---|---|
| CNCSI/I**A**AKAA | 10 (3.7%) | 4 (1.7%) | 0 (0%) | 14 (1.9%) |
| CNCSI/I**A**AKA**S** | 1 (0.4%) | 0 (0%) | 0 (0%) | 1 (0.1%) |
| CNCSI/I**AG**KAA | 37 (13.8%) | 11 (4.7%) | 8 (3.7%) | 56 (7.8%) |
| CNCSI/I**AG**KA**S** | 3 (1.1%) | 0 (0%) | 0 (0%) | 3 (0.4%) |
| CNCSI/I**F**AKA**S** | 1 (0.4%) | 1 (0.4%) | 0 (0%) | 2 (0.3%) |
| CNCSI/ISAKAA | 1 (0.4%) | 1 (0.4%) | 0 (0%) | 2 (0.3%) |
| CNCSI/ISAKA**S** | 1 (0.4%) | 1 (0.4%) | 0 (0%) | 2 (0.3%) |
| CNCSI/IS**G**KAA | 32 (11.9%) | 7 (3.0%) | 7 (3.2%) | 46 (6.4%) |
| CNCSI/IS**G**KA**S** | 2 (0.7%) | 0 (0%) | 0 (0%) | 2 (0.3%) |
| CNCSI/**VAG**K**GS** | 0 (0%) | 1 (0.4%) | 0 (0%) | 1 (0.1%) |
| C**I**CSI/IS**G**KAA | 1 (0.4%) | 0 (0%) | 0 (0%) | 1 (0.1%) |
| CNC**N**I/I**A**AKAA | 1 (0.4%) | 0 (0%) | 0 (0%) | 1 (0.1%) |
| CNC**N**I/I**AG**KAA | 4 (1.5%) | 1 (0.4%) | 0 (0%) | 5 (0.7%) |
| CNC**N**I/IS**G**KAA | 2 (0.7%) | 0 (0%) | 0 (0%) | 2 (0.3%) |
| C**I**C**N**I/I**AG**KAA | 8 (3.0%) | 2 (0.9%) | 0 (0%) | 10 (1.4%) |
| C**I**C**N**I/IS**G**KAA | 3 (1.1%) | 1 (0.4%) | 0 (0%) | 4 (0.6%) |
| C**IR**SI/I**A**AKAA | 1 (0.4%) | 0 (0%) | 0 (0%) | 1 (0.1%) |
| CN**RN**I/I**A**AKAA | 9 (3.3%) | 1 (0.4%) | 1 (0.5%) | 11 (1.5%) |
| CN**RN**I/I**A**AKA**S** | 1 (0.4%) | 1 (0.4%) | 0 (0%) | 2 (0.3%) |
| CN**RN**I/I**AG**KAA | 15 (5.6%) | 9 (3.8%) | 2 (0.9%) | 26 (3.6%) |
| CN**RN**I/I**AG**KA**S** | 3 (1.1%) | 2 (0.9%) | 2 (0.9%) | 7 (1.0%) |
| CN**RN**I/ISAKAA | 4 (1.5%) | 1 (0.4%) | 0 (0%) | 5 (0.7%) |
| CN**RN**I/ISAKA**S** | 1 (0.4%) | 0 (0%) | 0 (0%) | 1 (0.1%) |
| CN**RN**I/IS**G**KAA | 9 (3.3%) | 8 (3.4%) | 4 (1.9%) | 21 (2.9%) |
| CN**RN**I/IS**G**KA**S** | 0 (0%) | 1 (0.4%) | 0 (0%) | 1 (0.1%) |

| *Pfdhfr/Pfdhps* | 2010-2012 (N=269) | 2018 (N=235) | 2020 (N=216) | Total (N=720) |
|---|---|---|---|---|
| C**IRN**I/I**A**AKAA | 21 (7.8%) | 15 (6.4%) | 4 (1.9%) | 40 (5.6%) |
| C**IRN**I/I**A**AKA**S** | 1 (0.4%) | 1 (0.4%) | 0 (0%) | 2 (0.3%) |
| C**IRN**I/I**AG**KAA | 44 (16.4%) | 88 (37.4%) | 97 (44.9%) | 229 (31.8%) |
| C**IRN**I/I**AG**KA**S** | 6 (2.2%) | 15 (6.4%) | 18 (8.3%) | 39 (5.4%) |
| C**IRN**I/I**AG**K**G**A | 0 (0%) | 0 (0%) | 2 (0.9%) | 2 (0.3%) |
| C**IRN**I/I**AG**K**GS** | 0 (0%) | 0 (0%) | 2 (0.9%) | 2 (0.3%) |
| C**IRN**I/I**F**AKA**S** | 0 (0%) | 0 (0%) | 2 (0.9%) | 2 (0.3%) |
| C**IRN**I/I**FG**KAA | 1 (0.4%) | 1 (0.4%) | 0 (0%) | 2 (0.3%) |
| C**IRN**I/I**FG**KA**S** | 0 (0%) | 0 (0%) | 1 (0.5%) | 1 (0.1%) |
| C**IRN**I/ISAKAA | 9 (3.3%) | 2 (0.9%) | 0 (0%) | 11 (1.5%) |
| C**IRN**I/ISAKA**S** | 2 (0.7%) | 2 (0.9%) | 2 (0.9%) | 6 (0.8%) |
| C**IRN**I/ISAK**GS** | 0 (0%) | 0 (0%) | 2 (0.9%) | 2 (0.3%) |
| C**IRN**I/IS**G**KAA | 34 (12.6%) | 56 (23.8%) | 55 (25.5%) | 145 (20.1%) |
| C**IRN**I/IS**G**KA**S** | 1 (0.4%) | 2 (0.9%) | 0 (0%) | 3 (0.4%) |
| C**IRN**I/**VAG**KA**S** | 0 (0%) | 0 (0%) | 1 (0.5%) | 1 (0.1%) |
| C**IRN**I/**VAG**K**GS** | 0 (0%) | 0 (0%) | 6 (2.8%) | 6 (0.8%) |

**Supplementary Table 4:** Data on the prevalence of mutations at *Pfdhfr* and *Pfdhps* codons from previous and present studies conducted between 2009 and 2023 in Burkina Faso (N = number of samples analysed).

| **Year of sample collection** | **Sites** | **Population** | ***Pfdhfr* (Prevalence of mutations in %)** | | | | | ***Pfdhps* (Prevalence of mutations in %)** | | | | | | | | **Pfdhfr/ Pfdhps** | **References** |
|---|---|---|---|---|---|---|---|---|---|---|---|---|---|---|---|---|---|
| | | | **N51I (N)** | **C59R (N)** | **S108N (N)** | **I164L (N)** | **IRN (N)** | **I431V (N)** | **S436A/F (N)** | **A437G (N)** | **K540E (N)** | **A581G (N)** | **A613S (N)** | **GE (N)** | **VAGKGS/ VAAKGS (N)** | **IRN-GE (N)** | |
| **Before SMC* implementation** | | | | | | | | | | | | | | | | | |
| **2009** | **Bourasso** | Children | 50.2 (249) | 45.8 (249) | 51.4 (249) | NA | 35.3 (249) | NA | 84.5 (245) | 77.6 (245) | NA | NA | NA | NA | NA | NA | [1] |
| **2009** | **Satiri, Balla, and Kadomba** | Children | 58.1 (261) | 54.8 (261) | 55.0 (261) | 0 (261) | NA | NA | 35.1 (261) | 56.8 (261) | 0 (261) | NA | NA | NA | NA | NA | [2] |
| **2010** | **Nanoro** | Pregnant | 12.2 (255) | 61.2 (255) | 55.7 (255) | 0 (255) | 11.4 (255) | NA | NA | 34.2 (231) | 0 (231) | NA | NA | NA | NA | NA | [3] |
| **2010** | **Bobo-Dioulasso** | Pregnant | 41.2 (34) | 52.9 (34) | 64.7 (34) | 0 | 29.4 (34) | NA | NA | 79.4 (34) | 0 (34) | NA | NA | 0 (34) | NA | NA | [4] |
| **2010** | **Bobo-Dioulasso** | Pregnant | 71.3 (101) | 42.6 (101) | 64.4 (101) | 0 | 25.7 (101) | NA | NA | 80.2 (101) | 0 (101) | NA | NA | NA | NA | NA | [5] |
| **2010** | **Bourasso** | Children | 55.6 (223) | 62.8 (223) | 59.2 (223) | NA | 48.9 (223) | NA | 76.8 (207) | 72.9 (207) | NA | NA | NA | NA | NA | NA | [1] |
| **2010-2011** | **Ziniare** | Pregnant | 49.1 (311) | 50.4 (311) | 57.8 (311) | 0 (311) | 44.9 (311) | NA | 77.7 (311) | 75.3 (311) | 0 (311) | 0 (311) | 24.0 (311) | NA | NA | NA | [6] |
| **2011** | **Bourasso** | Children | 68.5 (213) | 72.8 (213) | 69.0 (213) | NA | 57.3 (213) | NA | 74.8 (222) | 80.2 (222) | NA | NA | NA | NA | NA | NA | [1] |
| **2010-2012** | **Nanoro** | Children and Adults | 48.9 (280) | 59.7 (280) | 65.7 (280) | 0 (280) | 43.6 (280) | 0 (280) | 61.8 (280) | 76.4 (280) | 0 (280) | 0 (280) | 8.2 (280) | 0 (280) | 0 (280) | 0 (269) | Our study |
| **2012** | **Bourasso** | Children | 80.6 (36) | 88.9 (36) | 88.9 (36) | NA | 69.4 (36) | NA | 88.6 (79) | 82.3 (79) | | NA | NA | NA | NA | NA | [1] |
| **2012** | **Bobo-Dioulasso** | Children and Adults | 60.9 (243) | 53.7 (242) | 63.8 (243) | 0 (242) | 44.3 (242) | NA | NA | 64.0 (237) | 0 (237) | NA | NA | 0 (237) | NA | 0 (237) | [7] |
| **2014-2015** | **Nanoro** | Pregnant | 63.9 (380) | 71.7 (380) | 74.3 (380) | NA | 61.1 (380) | NA | 70.3 (359) | 78.4 (359) | 0 (359) | NA | NA | 0 (359) | NA | 0 (353) | [8] |
| | **Nanoro** | Delivery | 72.7 (182) | 83.2 (182) | 86.4 (182) | NA | 70.5 (182) | NA | 72.1 (175) | 86.8 (175) | 1.1 (175) | NA | NA | 1.1 (175) | NA | 1.2 (168) | |
| | **Nanoro** | General population | 77.6 (355) | 83.2 (355) | 86.5 (355) | NA | 73.9 (355) | NA | 78.9 (352) | 84.8 (352) | 1.1 (352) | NA | NA | 1.1 (352) | NA | 0.9 (336) | |
| **After SMC implementation** | | | | | | | | | | | | | | | | | |
| **2016** | **Koupela** | Children | 92.9 (161) | 98.3 (161) | 98.9 (161) | NA | 92.4 (161) | 0 (161) | 70.5 (161) | 97.5 (161) | 0 (161) | 1.1 (161) | 5.1 (161) | 0 (161) | 0 (161) | 0 (161) | [9] |
| | **Koupela** | Adult | 98.3 (65) | 100 (65) | 100 (65) | NA | 98.1 (65) | 0 (65) | 79.9 (65) | 97.9 (65) | 0 (65) | 0 (65) | 3.3 (65) | 0 (65) | 0 (65) | 0 (65) | |
| **2018** | **Koupela** | Children | 83.1 (73) | 93.5 (73) | 97.4 (73) | NA | 73.9 (73) | 0 (73) | 55.3 (73) | 71.5 (73) | 0 (73) | 0 (73) | 10.9 (73) | 0 (73) | 0 (73) | 0 (73) | |

| | | | | | | | | | | | | | | | | | |
|---|---|---|---|---|---|---|---|---|---|---|---|---|---|---|---|---|---|
| | **Koupela** | Adult | 96.9 (106) | 100 (106) | 100 (106) | NA | 96.8 (106) | 0 (106) | 69.6 (106) | 94.4 (106) | 1.3 (106) | 0 (106) | 4.6 (106) | 0.5 (106) | 0 (106) | 0.5 (106) | |
| **2018** | **Nanoro** | Children and Adults | 78.6 (238) | 87.0 (238) | 88.6 (238) | 0 (238) | 77.3 (238) | 0.4 (236) | 65.3 (236) | 86.8 (236) | 0 (236) | 0.4 (236) | 11 (236) | 0 (236) | 0.4 (236) | 0 (235) | Our study |
| **2020** | **Nanoro** | Children and Adults | 89.4 (227) | 93.4 (227) | 93.4 (227) | 0 (227) | 89.4 (227) | 3.3 (216) | 67.6 (216) | 94.9 (216) | 0 (216) | 5.6 (216) | 16.6 (216) | 0 (216) | 2.8 (216) | 0 ((216) | Our study |
| **2021** | **Bobo-Dioulasso** | Children | 84.0 (128) | 100 (120) | 97.0 (106) | 0 (76) | NA | NA | 68 (119) | 88 (119) | 0 (137) | 1.0 (147) | 20.0 (138) | NA | NA | NA | [10] |

***Seasonal Malaria Chemoprevention**

## Reference


[1] Geiger C, Compaore G, Coulibaly B, Sie A, Dittmer M, Sanchez C, et al. Substantial increase in mutations in the genes pfdhfr and pfdhps puts sulphadoxine–pyrimethamine-based intermittent preventive treatment for malaria at risk in Burkina Faso. Trop Med Int Health 2014;19:690–7. https://doi.org/10.1111/tmi.12305.

[2] Somé AF, Zongo I, Compaoré Y-D, Sakandé S, Nosten F, Ouédraogo J-B, et al. Selection of Drug Resistance-Mediating Plasmodium falciparum Genetic Polymorphisms by Seasonal Malaria Chemoprevention in Burkina Faso. Antimicrob Agents Chemother 2014;58:3660–5. https://doi.org/10.1128/AAC.02406-14.

[3] Tahita MC, Tinto H, Erhart A, Kazienga A, Fitzhenry R, VanOvermeir C, et al. Prevalence of the dhfr and dhps Mutations among Pregnant Women in Rural Burkina Faso Five Years after the Introduction of Intermittent Preventive Treatment with Sulfadoxine-Pyrimethamine. PLoS ONE 2015;10:e0137440. https://doi.org/10.1371/journal.pone.0137440.

[4] Cissé M, Awandare GA, Somé FA, Hayette M-P, Guiguemdé RT. High concordance of Pfdhfr and Pfdhps genotypes between matched peripheral and placental isolates of delivered women in Bobo-Dioulasso, Burkina Faso. Ann Parasitol 2017;63:111–6. https://doi.org/10.17420/ap6302.93.

[5] Cisse M, Awandare GA, Soulama A, Tinto H, Hayette M-P, Guiguemdé RT. Recent uptake of intermittent preventive treatment during pregnancy with sulfadoxine-pyrimethamine is associated with increased prevalence of Pfdhfr mutations in Bobo-Dioulasso, Burkina Faso. Malar J 2017;16:38. https://doi.org/10.1186/s12936-017-1695-1.

[6] Coulibaly SO, Kayentao K, Taylor S, Guirou EA, Khairallah C, Guindo N, et al. Parasite clearance following treatment with sulphadoxine-pyrimethamine for intermittent preventive treatment in Burkina-Faso and Mali: 42-day in vivo follow-up study. Malar J 2014;13:41. https://doi.org/10.1186/1475-2875-13-41.

[7] Somé AF, Sorgho H, Zongo I, Bazié T, Nikiéma F, Sawadogo A, et al. Polymorphisms in K13, pfcrt, pfmdr1, pfdhfr, and pfdhps in parasites isolated from symptomatic malaria patients in Burkina Faso. Parasite n.d.;23:60. https://doi.org/10.1051/parasite/2016069.

[8] Ruizendaal E, Tahita MC, Geskus RB, Versteeg I, Scott S, d'Alessandro U, et al. Increase in the prevalence of mutations associated with sulfadoxine–pyrimethamine resistance in Plasmodium falciparum isolates collected from early to late pregnancy in Nanoro, Burkina Faso. Malar J 2017;16:179. https://doi.org/10.1186/s12936-017-1831-y.

[9] Beshir KB, Muwanguzi J, Nader J, Mansukhani R, Traore A, Gamougam K, et al. Prevalence of Plasmodium falciparum haplotypes associated with resistance to sulfadoxine–pyrimethamine and amodiaquine before and after upscaling of seasonal malaria chemoprevention in seven African countries: a genomic surveillance study. Lancet Infect Dis 2022;0. https://doi.org/10.1016/S1473-3099(22)00593-X.
[10] Roh ME, Zongo I, Haro A, Huang L, Somé AF, Yerbanga RS, et al. Seasonal Malaria Chemoprevention Drug Levels and Drug Resistance Markers in Children With or Without Malaria in Burkina Faso: A Case-Control Study. J Infect Dis 2023;228:926–35. https://doi.org/10.1093/infdis/jiad172.

**Supplementary Table 5**: Heterogeneity measurement at each codon of studies included in the meta-analysis

| Codons | $I^2$ Statistic | Cochran's Q test | p-value | Conclusion |
|---|---|---|---|---|
| **N51I** | 0 | 4.9723 | 0.99973 | No heterogeneity |
| **C59R** | 0 | 4.9884 | 0.99972 | No heterogeneity |
| **S108N** | 0 | 5.0352 | 0.99970 | No heterogeneity |
| **S436A/F** | 0 | 2.4151 | 0.99996 | No heterogeneity |
| **A437G** | 0 | 3.9714 | 0.99995 | No heterogeneity |
